# Patients’ beliefs regarding informed consent for low-risk pragmatic trials

**DOI:** 10.1186/s12874-017-0424-3

**Published:** 2017-09-18

**Authors:** Rafael Dal-Ré, Antonio J. Carcas, Xavier Carné, David Wendler

**Affiliations:** 1 Clinical Research, BUC (Biosciences UAM+CSIC) Program, International Campus of Excellence, Universidad Autónoma de Madrid, Ciudad Universitaria de Cantoblanco, Einstein 3, 28049 Madrid, Spain; 2 Chair on Bioethics “Grifols Foundation”, University of Vic - Central University of Catalonia, Miquel Martí i Pol 1, Campus Miramarges, E-08500, Vic, Barcelona, Spain; 3 Clinical Pharmacology Department, La Paz University Hospital, IdiPaz, School of Medicine, Universidad Autónoma de Madrid, Paseo de la Castellana 261, 28046 Madrid, Spain; 4 Clinical Pharmacology Department, Clínic Hospital, August Pi i Sunyer Biomedical Research Institute (IDIBAPS); Clinical Fundamentals Department, Universidad de Barcelona, Carrer de Villarroel 170, 08036 Barcelona, Spain; 5 Section on Research Ethics, Department of Bioethics, NIH Clinical Center, 10 Center Dr, Bethesda, MD 20814 USA; 6 Epidemiology Unit, Health Research Institute-Fundación Jiménez Díaz University Hospital, Universidad Autónoma de Madrid, Avda. Reyes Católicos 2, E-28040 Madrid, Spain

**Keywords:** Survey, Low-risk pragmatic trials, Written informed consent, Verbal informed consent, General notification, Clinical trials regulation, Low-intervention clinical trials

## Abstract

**Background:**

The requirement to obtain written informed consent may undermine the potential of pragmatic randomized clinical trials (pRCTs) to improve evidence-based care. This requirement could compromise trials statistical power or even force it to close them down prematurely. However, recent data from the U.S. and Spain suggest that a majority of the public endorses written consent for low-risk pRCTs. The present manuscript assesses whether this view is shared by patients.

**Methods:**

This was a cross-sectional, probability-based survey, with a 2 × 2 factorial design, assessing support for written informed consent versus verbal consent or general notification for two low-risk pRCTs in hypertension, one comparing 2 drugs with similar risk/benefit profiles and the other comparing the same drug being taken in the morning or at night. This web-based survey was conducted in May 2016. Two-thousand and eight adults who were representative of the Spanish population participated in the survey (response rate: 61%). Of these 2008 respondents, 338 indicated that they had been diagnosed with hypertension and were being treated with prescription medicines for this condition at the time of responding to the survey. The primary outcome measures were respondents’ personal preference and recommendation to a research ethics committee regarding the use of written informed consent versus verbal consent or general notification.

**Results:**

Overall, 74% of the 338 patient respondents endorsed written consent. In both scenarios, general notification received significantly more support (30.6%-44.7%) than verbal consent (13.3%-17.6%). 43% of respondents preferred and/or recommended general notification rather than written consent.

**Conclusions:**

As in the survey of the general public, more patients endorsed written consent than the alternative option. However, two factors suggest that a different approach to written consent should be investigated for low-risk pRCTs: a) a substantial minority of respondents supported general notification, b) data from the US have shown that most patients who prefer written consent are willing to forego it if obtaining written consent makes the trial too difficult to be conducted; and c) 2016 CIOMS guidelines endorse waivers of consent when the trial fulfills specific conditions. Surveys in other EU countries are needed to assess what patients believe towards pRCTs. If similar results to that reported in this study are found, it is foreseeable that with educational efforts, general notification could be an acceptable and widespread approach to the conduct of low-risk pRCTs.

**Electronic supplementary material:**

The online version of this article (10.1186/s12874-017-0424-3) contains supplementary material, which is available to authorized users.

## Background

Recruiting a sufficient number of participants is a common problem for clinical trials. Insufficient recruitment can reduce statistical power, waste resources, increase costs and even result in the premature termination of a trial [1–3]. Acknowledging that appropriately informing potential trial participants is a key ethical principle in clinical research, seeking written informed consent could jeopardize the conduct of clinical trials. In response, a number of studies have assessed if modifications to the consent form and/or the consent process could ease participants’ recruitment [4]. On the other hand, there is scarce empirical evidence of what type of information potential research participants want to know about the study: for instance, only 39% and 76% of potential participants wanted to be told about voluntariness and the purpose of the study, respectively [5].

Currently, in both the US and EU clinical trials regulations, and for all types of trials, except for cluster-randomized trials, written informed consent is asked from all participants. This requirement creates huge challenges to many pragmatic randomized controlled trials (pRCTs) preventing the appropriate conduct of many of them due to insufficient or biased recruitment [6]. Research ethics committees (RECs) could adapt current informed consent requirements to the specific needs of the research, as was the case in two pRCTs conducted in the UK with commonly prescribed medications where short (2-page) participants information sheets where used to inform potential participants [7]. However, with current regulations, RECs could never change to a verbal consent, or waived participant’s consent in the conduct of pRCTs assessing the comparative effectiveness of commercially available medications. These two alternatives to written informed consent (verbal consent or general notification, i.e., a non-specific informed consent approach), however, have been shown to be supported by substantial minorities of the general public in the US and Spain when being asked on hypothetical low-risk pRCTs with commonly prescribed drugs [8,9]. It is important to understand whether having a chronic condition might influence individual views on written informed consent for low-risk pRCTs, of special interest since this type of trial will be frequently conducted for the assessment of commonly prescribed drugs.

## Methods

Two thousand and eight adults participated in a survey conducted in Spain in May 2016, that replicated a previous survey that was conducted in the US [8]. The Spanish survey was administered to individuals belonging to Netquest (GfK group) panel (https://www.netquest.com/es/home/encuestas-online-investigacion). This panel comprises almost 200.000 people. Adult Spaniards with internet access are invited to join (‘single-use’ invitation) with the goal of ensuring a representative sample of the non-institutionalized civilian Spanish population. This was a probability-based online panel –except for the oldest (≥75 years) age group which is less represented than in the general population. The design, conduct and results of the survey have been explained in detail elsewhere [9].

The survey used a cross-sectional, 2 × 2 factorial design (Table 1). The survey started by explaining a hypothetical hospital in which all patients were informed through letters, brochures and posters on the simultaneous provision of care and the conduct of research (Additional file 1). Two hypothetical scenarios were assessed: two low-risk pRCTs in hypertension, comparison of 2 drugs with similar risk/benefit ratio or taking the same drug in the morning or at night. Each scenario had two routes: written consent vs verbal consent; written consent vs general notification. Each respondent was randomized to one of the 4 routes.

**Table 1:** Experimental design of the survey (Modified from Nayak et al. [8])

Research conducted at the time to providing health care	Hospitals that integrate research as part of care provision Patients informed that studies are conducted through letters, posters, and brochures All studies are reviewed and approved by a REC, which comprises researchers, clinicians, ethicists, patient representatives, and community members			
High blood pressure	Affects millions of persons in Spain Can lead to stroke, heart attack, and/or kidney disease if untreated			
Pragmatic RCT scenario	*Scenario 1: Drug “CTD” or “TRT”?* Two Health Authorities-approved medicines Both effective in lowering high blood pressure; similar adverse effects Unknown which is more effective		*Scenario 2: Dose timing, “morning” or “night”?* Patients told to take medicine at same time each day Unknown whether morning or night more effective	
Trial proposal	Random assignment to CTD or TRT Patient’s medicine can be changed at any time by patient or physician		Random assignment of whether told to take medicine at morning or night Patient’s medicine can be changed at any time by patient or physician	
Debate	REC is debating the best way to get consent for this study			
*Consent options*	*Written consent* vs. *verbal consent*	*Written consent* vs. *general notification*	*Written consent* vs. *verbal consent*	*Written consent* vs. *general notification*
Written consent	• Some members argue patients should give study-specific written consent • Consent form would include purpose, risks and benefits, alternatives, method of maintaining privacy, and contact information; participation would be voluntary • Written consent would require extra time and effort • In some cases, if written consent is required, studies may not be done			
Alternative option	*General Notification* • Other members argue that because the risks are low, general notification through posters, brochures, and letters is enough • Eligible patients would be automatically enrolled without being informed *Verbal Consent* • Other members argue that because the risks are low, verbal consent is enough • Patient’s physician would briefly explain the study			

Shows the 2 × 2 factorial design and information presented to respondents. Half received a drug RCT scenario comparing 2 first-line drugs; the others received a dose-timing RCT scenario comparing morning vs. night dosing. Half of participants in each group chose between written consent and general notification; the rest chose between written consent and verbal consent. *CTD* chlorthalidone, *RCT* randomized, controlled trial; *REC* Research ethics committee, *TRT* hydrochlorothiazide

In the hypothetical scenarios presented to respondents, the primary outcome measures were the respondent’s recommendation to the REC (“If you were to give advice to the REC, would you recommend written consent or general notification/verbal consent?”) and the respondent’s preference (“If you were a patient in this hospital, which would you personally prefer, written consent or general notification/verbal consent?”). Responses to both questions were ‘definitely’ or ‘probably’ for both written consent and the alternative option.

Respondents were asked to evaluate the trial by indicating whether they agree, using a 7-point scale (1 = strongly disagree, 7 = strongly agree), with the following three statements: a) “It is valuable to study whether one treatment option is more effective than the other for treating high blood pressure”; b) “Patients who participate in the randomized trial face greater risks than patients who receive usual care”; and c) “Patients who participate in the randomized trial are more likely to improve (lower) their high blood pressure than patients who receive usual care”.

Since both of the theoretical pRCTs involved hypertension, participants were asked to report on whether they have been diagnosed with hypertension and whether they were receiving treatment with prescription medications. This article reports on the results obtained in the 338 respondents who indicated that they had been diagnosed with hypertension and were being treated at the time of responding to the survey.

### Statistical analysis

Recommendations to the REC and personal preferences for written consent or the alternative approach were dichotomized. Logistic regression models were used to assess whether the pRCT scenario and alternative consent/notification option were associated with respondents’ recommendations and personal preferences. The models included main effects for the research scenario (drug pRCT vs. dose-timing pRCT) and the alternative option (general notification; verbal consent), as well as the interaction of the 2 factors. To evaluate the association between respondents’ perceptions of the study’s value, risk, and benefit and support for the alternative option, the Pearson chi-square test of independence corrected for bootstrap was used.

All analyses were conducted in IBM SPSS statistics, version 21. According to final sample distribution, post-stratification weights were not used. Statistical significance was defined as a *P* value less than 0.05, and all tests were 2-sided.

## Results

**Fig. 1:**
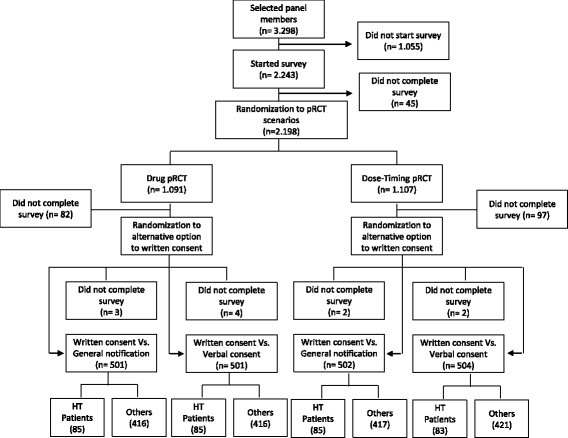
Study flow diagram. HT: hypertensive

The survey was forwarded to 3298 panel members and started by 2243, of which 45 dropped out before they were randomized to one of the two pRCTs scenarios. After randomization, 179 were excluded for nonresponses to one of the two alternative options (written consent vs general notification or written consent vs verbal consent). Finally, 11 individuals were excluded for not responding to both primary outcomes (recommendation to the REC and personal preference), leaving 2008 panelists completers (response rate: 60.9%). The 338 respondents who indicated that they had been diagnosed with hypertension and were being treated with prescription medicines for this condition at the time of the survey (Fig. 1), were almost evenly distributed into the 4 groups and did not show statistically significant differences in any of the assessed characteristics (Table 2). Some 35% of these 338 respondents were ≥65 years old, quite different from the Spanish hypertensive population, of which 49% belong to this age group [10].

**Table 2:** Characteristics of the 338 hypertensive respondents by scenario and group

	Overall (*n* = 338) n (%)		Drug RCT, %		Dose-Timing RCT, %	
			Written consent vs General Notification (*n* = 85)	Written Consent vs Verbal Consent (*n* = 85)	Written consent vs General Notification (*n* = 85)	Written Consent vs Verbal Consent (*n* = 83)
Age						
18/44 y	50	(14.8)	18.8	8.2	12.9	19.3
45/64 y	169	(50.0)	50.6	55.3	48.2	45.8
≧65 y	119	(35.2)	30.6	36.5	38.8	34.9
*p* = 0.377						
Sex						
Male	201	(59.5)	58.8	63.5	61.2	54.2
Female	137	(40.5)	41.2	36.5	38.8	45.8
*p* = 0.648						
Geographical area						
North	48	(14.2)	9.4	15.3	14.1	18.1
Northeast	60	(17.8)	21.2	16.5	11.8	21.7
East	46	(13.6)	12.9	14.1	15.3	12.0
Central-West	94	(27.8)	34.1	27.1	29.4	20.5
South	65	(19.2)	15.3	21.2	21.2	19.3
Islands	25	(7.4)	7.1	5.9	8.2	8.4
*p* = 0.808						
Marital status						
Never married	31	(9.2)	11.8	5.9	7.1	12.0
Married or living with partner	251	(74.3)	74.1	77.6	77.6	67.5
Other	56	(16.6)	14.1	16.5	15.3	20.5
*p* = 0.579						
Annual Household income						
< 12.600 €	65	(19.2)	22.3	15.3	20.1	19.3
12.600 - 25.000€	90	(26.6)	31.8	27.1	29.4	18.1
25.001 – 38.000€	56	(16.6)	10.6	18.8	18.8	18.1
> 38.000€	53	(15.7)	14.1	17.6	14.1	16.8
No answer	74	(21.9)	21.2	21.2	17.6	27.7
*p* = 0.654						
Employment status						
Employed	106	(31.4)	31.8	36.4	24.7	32.5
Unemployed or other	111	(32.8)	34.1	22.4	43.5	31.4
Retired	121	(35.8)	34.1	41.2	31.8	36.1
*p* = 0.174						
Education						
Primary school	77	(22.8)	23.5	21.2	21.2	25.3
Secondary education	105	(31.1)	31.8	24.7	36.5	31.3
High school	101	(29.9)	28.2	32.9	31.8	26.5
College and postgraduate	55	(16.3)	16.5	21.2	10.6	16.9
*p* = 0.731						
Religious attendance						
Regularly	59	(17.5)	18.8	17.6	14.1	19.3
Rarely	59	(17.5)	23.5	16.5	16.5	13.3
Never	177	(52.4)	44.7	57.6	55.3	51.8
No answer	43	(12.7)	12.9	8.2	14.1	15.7
*p* = 0.625						
Ideology						
1 -2 Left	63	(18.6)	17.6	20.0	14.1	22.8
3	67	(19.8)	21.3	20.0	17.7	20.5
4 Moderate	105	(31.1)	29.4	37.6	34.1	22.9
5-6-7 Right	57	(16.9)	14.1	15.3	17.6	20.5
No answer	46	(13.6)	17.6	7.1	16.5	13.3
*p* = 0.541						
RCT: Randomized, controlled trial.						

### Recommendations to the REC and personal preferences

**Fig. 2:**
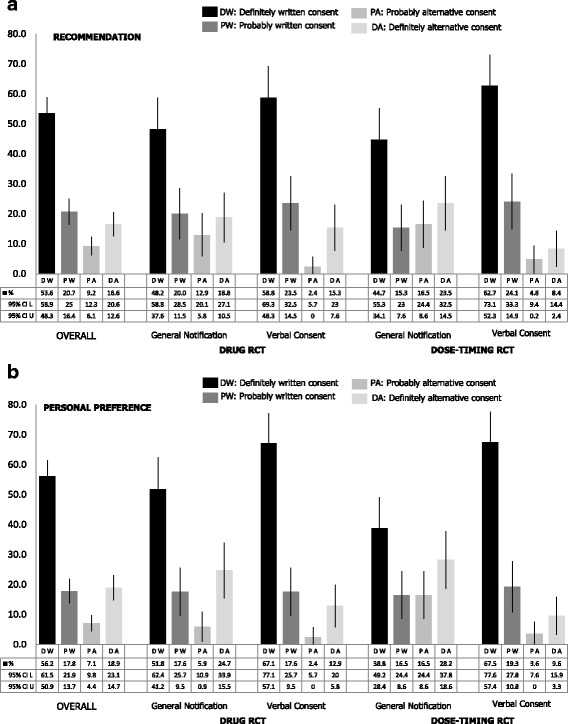
Recommendations to the research ethics committee (top) and personal preferences (bottom) for written consent and the alternative option. 2**a**.-Recommendation ro research ethics committee. 2**b**.-Personal preference. CI: Confidence interval; L: Lower limit; U. Upper limit; RCT: Randomized controlled trial

Overall, 74.3% of all the respondents would definitely or probably recommend use of written consent to the REC (Fig. 2a). In the drug pRCT, 31.8% would recommend general notification, whereas 17.6% would recommend verbal consent. In the dose-timing pRCT, 40.0% would recommend general notification, whereas 13.3% would recommend verbal consent instead of written consent.

**Fig. 3:**
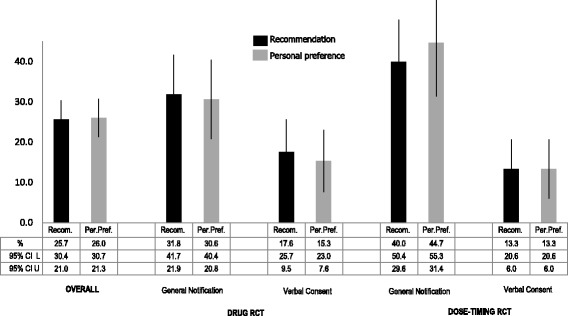
Support for alternative options to written consent. CI: Confidence interval; L: Lower limit; U. Upper limit; RCT: Randomized controlled trial

Overall, 74% of all respondents would definitely or probably prefer the use of written consent (Fig. 2b). In the drug pRCT, 30.6% prefer general notification, whereas 15.3% prefer verbal consent. In the dose-timing pRCT, 44.7% prefer general notification, whereas 13.3% prefer verbal consent instead of written consent. Fig. 3 shows respondents’ recommendations to the REC and their personal preferences.

Considering only those 170 respondents who were presented with the option of written consent or general notification in the drug pRCT and the dose-timing pRCT scenarios, 42.9% preferred and/or recommended general notification to the REC. Among the 168 respondents who were presented with the option of verbal consent or written consent, 16.1% preferred and/or recommended verbal consent.

Responses to the 2 items were consistent across the groups, with most (from 87.1% to 100%) having the same recommendation and personal preference (Table 3). In the dose-timing pRCT consistency is statistically significantly greater (100% vs 88.3%; *p* = 0.001) when verbal consent is the alternative option instead of general notification. In both scenarios (drug RCT and dose-timing RCT) the percentage of respondents who preferred and/or recommended the alternative option is statistically significantly higher when the alternative is general notification rather than verbal consent (37.6% vs 18.8%, *p* = 0.005, in drug RCT; and 48.2% vs 17.3%, *p* < 0.001 in dose-timing RCT). The percentage of respondents who preferred the alternative option was not statistically significantly different in any of the 4 groups compared with the percentage of respondents that would recommend the alternative option to the REC (Fig. 3).

**Table 3:** Cross tabulation of respondents’ recommendation to the research ethics committee (REC) and personal preferences

*Variable*	*Overall, %* *(n = 338)*	*Drug pRCT,* *%*		*Dose-timing pRCT,* *%*	
		Written consent vs General notification (*n* = 85)	Written consent vs Verbal consent (*n* = 85)	Written consent vs General notification (*n* = 85)	Written consent vs Verbal consent (*n* = 83)
Recommended written consent, preferred written consent	70.4	62.4	81.2	51.8	86.7
Recommended written consent, preferred alternative option	3.8	5.9	1.2	8.2	0.0
Recommended alternative option, preferred written consent	3.6	7.1	3.5	3.5	0.0
Recommended alternative option, preferred alternative option	22.2	24.7	14.1	36.5	13.3
		*p* = 0.042		*p* < 0.001	
Same personal preference and recommendation to the REC (Consistent responses)	92.6	87.1	95.3	88.3	100.0
		*p* = 0.051		*p *= 0.001	
Personal preference and/or recommendation alternative option	92.6	37.6	18.8	48.2	17.3
		*p* = 0.005		*p* < 0.001	

*pRCT* pragmatic randomized controlled trial

A logistic regression model was used to test the effect of the experimental design of the survey on recommendations for using the alternative option over written consent. As mentioned above, the main effect is the alternative option presented: in both scenarios, drug pRCT and dose-timing pRCT, a statistically significant higher percentage of respondents recommended and preferred the alternative option of general notification rather than verbal consent (*p* < 0.001). The likelihood to recommend general notification to the REC is close to 4 times higher than verbal consent in the dose-timing pRCT scenario (OR = 3.712; *p* < 0.001) and almost 3 times higher in the drug pRCT scenario (OR = 2.910, *p* < 0.001). Similarly, the likelihood of preferring general notification is close to 4 times higher than verbal consent in the dose-timing pRCT scenario (OR = 3.974; *p* < 0.001) and almost 3 times higher in the drug pRCT scenario (0R = 2.724, *p* < 0.001). A table with the logistic regression analysis is shown in Additional file 2.

### Views of pragmatic RCT scenarios

A large majority of respondents agreed that the described trial was valuable, with no statistically significant differences between the two scenarios: 90.6% in drug pRCT, 93.5% in dose-timing pRCT (Table 4). Some 36% and 48% of respondents in both scenarios believed that trial participants would face greater risks and greater potential benefit, respectively, than those receiving usual care.

**Table 4:** Views of the hypertensive patients on statements about social value, risk and benefit of the pragmatic randomized controlled trial (pRCT) scenarios

*Statement*	*Scenario*	*Response, %*			*P value*
		Disagree	Neutral	Agree	
It is valuable to study whether one treatment option is more effective than the other for treating high blood pressure	Drug pRCT ^a^ Dose-timing pRCT ^b^	3.5 1.8	5.9 4.8	90.6 93.5	0.538
Patients who participate in the randomized trial face greater risks than patients who receive usual care	Drug pRCT ^a^ Dose-timing pRCT ^b^	31.8 40.5	27.6 25.6	40.6 33.9	0.233
Patients who participate in the randomized trial are more likely to improve (lower) their high blood pressure than patients who receive usual care.	Drug pRCT ^a^ Dose-timing pRCT ^b^	17.6 14.9	36.5 35.7	45.9 49.4	0.729

^a^ *n* = 170

^b^ *n* = 168

## Discussion

The majority (74%) of patients participating in this survey endorsed written informed consent for low-risk pRCTs. This finding is similar to the results found in the general population [9] (77% supported written consent) and suggests that being affected by the condition under study does not affect respondents’ beliefs regarding the need to obtain written informed consent for low-risk pRCTs. In particular, patients were not more willing to accept an alternative to written informed consent compared to the general population.

The responses observed in the Spanish general population [9] and in the patients were rather similar, although a few slightly differences were noted. Although the percentages of respondents who recommended and/or preferred the alternative option were similar in the general population (40%) and the hypertensive patients (43%), the patients were more likely to have consistent responses between their preference and their recommendation to the REC (22.2% versus 17.7%) [9]. Similarly, the hypertensive patients had somewhat worse understanding of the perceived risks and benefits of being enrolled in a clinical trial: 36% of hypertensives (vs 32% in the general population) thought participating in a RCT poses more risks than usual care, whereas 48% of hypertensive respondents (vs 43%) believed participating in a RCT offers greater potential benefits. The different age distribution (hypertensive patients sample being much older) might help to explain these two differences; this could be object of a future study.

These present results are somewhat surprising. Limited available data on what patients (120 respondents of an online survey) believed regarding consent to participate in a hypertension drug low-risk pRCT, found that only 38% of respondents endorsed written consent and 42% endorsed verbal consent. In contrast, 21% indicated that broad notification was sufficient (16%) or no notification (5%) was needed [11]. The relatively high percentage of respondents in our survey supporting general notification (43%) versus written consent may be explained, in part, by the high trust the Spanish population has in physicians (95%) and in the universal public National Health Service (75%) [12], where the hypothetical scenarios were placed.

To the best of our knowledge, this is the first study conducted in any EU Member State assessing the opinion of patients with regards to written informed consent for low-risk pRCTs. However, it has several important limitations. First, as with the parent survey [9], the response rate was 61%, and it is not possible to determine whether non-respondents might differ from respondents; in addition, framing effects and the use of hypothetical scenarios might have influenced respondents’ attitudes -notably, the hypothetical scenarios, involving pRCTs conducted in clinical settings, likely were unfamiliar to many respondents. Second, the age distribution of this study sample is quite different from that of the Spanish hypertensive population: whereas 49% of hypertensive patients in Spain are ≥65 years old [9], in this study only 35% were in this age group. Third, we relied on self-report for whether the respondents were diagnosed with hypertension and whether they were taking medication. Finally, the study design did not allow us to assess directly which alternative method (verbal consent or general notification) respondents would prefer or recommend.

The informed consent process is not “one size fits all” and should be tailored to context [13]. In some cases it can even be waived. Thus, the recently issued CIOMS guidelines endorse the waiver of participant’s informed consent when the three following conditions are satisfied: a) the research is not practicable without the waiver; b) the research has important value; and c) the research poses no more than minimal risk to participants; in any case, the relevant REC must approve the waiver of informed consent [14]. Many low-risk pRCTs could fulfill these three conditions since, a) requiring informed consent to participants might jeopardize its correct conduct, since it is a barrier to unselected participant recruitment [15]; b) when a trial helps policy makers determine which options to fund in National Healthcare Systems [16], the (public) value of the trial is out of question, and c) low-risk pRCTs typically pose no incremental risk (i.e., no more than minimal risk) compared to clinical care [17]. pRCTs that would easily fulfill these three criteria are those conducted with commercially available medications and using routing electronic health records, also known as point-of-care trials [7, 18–21]. The conduct of this type of pRCTs will most likely be fostered if investigators show to RECs the fulfillment of these three conditions. However, the EU new clinical trials regulations do not take into account the possibility of a waiver of the classical written informed consent except for cluster-RCTs [22].

It seems clear that to ensure the proper conduct and recruitment of participants to low-risk pRCTs there is a need to identify new approaches to written informed consent that should end-up with the amendment of current clinical trials regulations [23]. Two different approaches have been proposed in the US when considering low-risk pRCTs when clinical research is integrated in medical care: one supports asking for patient’s verbal consent to participate after explaining that randomization will decide participant’s treatment [24]; whereas the other strongly believes there is no need to ask for specific consent [25, 26].

## Conclusion

Future surveys are needed within the EU member states addressing patients’ beliefs with regards to informed consent in low-risk pRCTs and, very importantly, to know if their beliefs would change if they were aware that the trial could not be conducted as expected if participants’ written informed consent is sought. This is relevant since a majority of both US patients [11] and public [27] endorsing written or verbal consent for low-risk pRCTs changed their minds if consent poses huge difficulties to the conduct of such trials and would accept general or no notification. Now that the conduct of low-risk pRCTs with no participant’s consent could be ethically acceptable in certain circumstances [14], it would be appropriate to know what EU patients believe on this subject so regulators could know what the society they serve is expecting. However, it should be acknowledged that most (if not all) RECs in the EU will reject on legal grounds the approval of a low-risk pRCT asking for a waiver of participants’ consent until EU clinical trials regulation is appropriately amended.

## Additional files

Additional file 1: Survey Questionnaire (DOCX 33 kb)

Additional file 2: Recommendation to research ethics committee and personal preference: written consent or alternative option. Logistic regression (DOCX 14 kb)

Additional file 3: Anonymous participant level data (XLSX 50 kb)

## Abbreviations

CIOMS: Council for International Organizations of Medical Sciences (Geneva, Switzerland)
pRCT: Pragmatic randomized controlled trial
RCT: Randomized controlled trial
EU: European Union
