# Supplementary material for: Patients’ beliefs regarding informed consent for low-risk pragmatic trials
Source: BMC Med Res Methodol. 2017 Sep 18;17:145. doi: 10.1186/s12874-017-0424-3 (PMC5604493; doi:10.1186/s12874-017-0424-3)
Supplement: Supplementary file 1 — Survey Questionnaire (DOCX 33 kb) [file 12874_2017_424_MOESM1_ESM.docx]

**Dal-Ré, Carcas, Carné & Wendler**

**Additional file-1.**

**Survey Questionnaire**

[Panel members were randomized to receive either the drug RCT or dose timing RCT scenario and to receive either the general notification or the verbal consent option. The text in brackets indicates the arm of the survey and was not shown to the respondents]

**[Common information provided to all participants]**

“Obtaining informed consent from comparative effectiveness pragmatic clinical trials: survey to Spanish adult general population”

INTRODUCTION

For many years it is known that those hospitals conducting medical research use to provide better health care. Currently, some Spanish hospitals are learning how to integrate research with provision of health care. That’s why, hospital research teams conduct this type of comparative trials of two or more treatments as part of the health care. Patients who seek care at these hospitals are routinely informed by means of an informative letter, posters and brochures that are placed and provided in the hospitals.

Before a clinical trial is started, this must be approved by a research ethics committee whose job it is to protect the rights and well-being of patients. The results of the trial will be useful for future patients of the hospital where the trial is conducted and of other Spanish and foreign hospitals. In fact, sometimes, millions of patients benefit from the results of only one trial.

In this survey, we will describe an example of this type of clinical trial. This is related with the treatment of hypertension (high blood pressure),an area of special interest to improve patient care.

**What is hypertension?**

High blood pressure is a chronic medical condition that affects millions of Spaniards. If left untreated for many years, it can lead to a variety of medical problems, such as:

stroke

heart attacks

kidney diseases

**[Scenario 1, Drug randomized clinical trial]**

**Common Treatments for High Blood Pressure: CTD or TRT**

Although exercise and other lifestyle changes can help lower blood pressure, many people require treatment with medicine. The two most commonly recommended medicines for high blood pressure are CTD and TRT (we have changed the names but they refer to real drugs):

**-**both have been approved by the health authorities and have been used for many years to treat high blood pressure

**-**both are effective in lowering blood pressure

**-**both have similar, mostly non-serious side effects

Because **CTD** and **TRT** are so similar and have never been compared head-to-head, it is not known whether **CTD** or **TRT** is more effective than the other for treating high blood pressure.

Even if it turns out that one drug is only slightly better than the other, because so many people have high blood pressure, this knowledge could benefit the long-term health of millions of people.

**Clinical trial proposal**

To show what is the best of these medicines, a randomized clinical trial (this means that medicines will be randomized between participants) will be conducted. This means that when each patient enters the study a computer program will randomly decide (like the flip of a coin) whether the patient receives CTD or TRT. After one year, the patients who receive **CTD** will be compared to the patients who receive **TRT** to assess which medicine is better. The patient's medicine can be changed at any time if the patient or the patient’s doctor feels that a different medicine would be better.

**Recruiting Participants**

The research ethics committee is composed of physicians, nurses, individuals with no relation to health care and, at least, a lawyer and a patient representing the patients. Is tasked with overseeing all types of research conducted in the national health system. The research ethics committee is having a debate about the best way to conduct this trial with **CTD** and **TRT**..

Some members argue that patients should be given a **written consent** form about the study and asked to sign the form if they choose to participate. The consent form would **include information** about:

-the purpose of the research,

-risks and benefits,

-any alternative treatments,

-how patient privacy will be maintained,

-contact information for questions,

-a statement that participation is voluntary.

However, this written consent process will require extra time and effort from both the physician and the patient. As a result, requiring written consent would make it difficult to integrate research studies like this into routine health care practice. In some cases, if written consent is required, studies like these may not be done.

**[Option A; 1.1.General Information arm]**

Other members of the research ethics committee argue that written consent is unnecessarily burdensome in this case because the study has **very low risk**:

-CTD and TRT are both commonly used,

-have similar side effects, and

-doctors do not know which one is better.

These members of the research ethics committee recommend that it is enough to provide **general notification** to all patients (through posters, brochures, and letters) that the hospital conducts research like this. Eligible patients for the trial who wish to begin treatment for their high blood pressure would automatically be enrolled in the trial without being informed or specifically asked if they would like to participate. From the patients’ perspective, all other aspects of care they receive would be no different than usual, except for the fact that a computer randomly chooses the treatment.

Q1a. If you were to give advice to the research ethics committee, would you recommend Written Consent or General Notification?

| Written Consent | | General Notification | |
| --- | --- | --- | --- |
| Definitely | Probably | Probably | Definitely |
| 1 | 2 | 3 | 4 |

Q2a. If you were a patient in this hospital, which would you personally prefer, Written Consent or General Notification?

| Written Consent | | General Notification | |
| --- | --- | --- | --- |
| Definitely | Probably | Probably | Definitely |
| 1 | 2 | 3 | 4 |

**[Option B; 1.2. Verbal Consent arm]**

Other members of the research ethics committee argue that written consent is unnecessarily burdensome in this case because the study has **very low risk**:

-CTD and TRT are both commonly used,

-have similar side effects, and

-doctors do not know which one is better.

These members of the research ethics committee recommend that it is enough to obtain **verbal consent** from the patient. The patient’s doctor would briefly explain the study of CTD vs. TRT:

-explain that the two drugs are both approved by the health authorities and widely used medications,

-discuss their potential side effects, and

-emphasize that the selection will be random.

The doctor would ask whether the patient would like to participate in the trial, and then record the patient’s decision in the medical record. For those that participate, all other aspects of care they receive would be no different than usual, except for the fact that a computer randomly chooses the treatment.

Q1b. If you were to give advice to the ethics review board, would you recommend Written Consent or Verbal Consent?

| Written Consent | | Verbal Consent | |
| --- | --- | --- | --- |
| Definitely | Probably | Probably | Definitely |
| 1 | 2 | 3 | 4 |

Q2b. If you were a patient in this hospital, which would you personally prefer, Written Consent or Verbal Consent?

| Written Consent | | Verbal Consent | |
| --- | --- | --- | --- |
| Definitely | Probably | Probably | Definitely |
| 1 | 2 | 3 | 4 |

**[To all respondents of Scenario 1; 1.3. To both scenarios 1.1.and 1.2]**

To finalize, we would also like to ask some questions about your opinions on how this proposed clinical trial compares to usual care. As a reminder:

A.In the randomized trial the patient’s doctor will use a computer to randomly choose the treatment (CTD or TRT) for the patient.

B.In usual care the patient’s doctor will choose the treatment (CTD or TRT) for the patient.

In either case, the medication the patient is taking can be changed at any time if the patient or the patient’s doctor feels that a different medicine would be better.

Please rate the following statements on a scale from 1 (strongly disagree with the content of the sentence) to 7 (strongly agree).

| Strongly Disagree |  |  | I would not know to give an opinion |  |  | Strongly Agree |
| --- | --- | --- | --- | --- | --- | --- |
| 1 | 2 | 3 | 4 | 5 | 6 | 7 |

Q3a. It is valuable to study whether one treatment option is more effective than the other for treating high blood pressure.

Q4a. Patients who participate in the randomized trial face greater risks than patients who receive usual care.

Q5a. Patients who participate in the randomized trial are more likely to improve (lower) their high blood pressure than patients who receive usual care.

**We have concluded. Thanks for your collaboration**

**[Scenario 2, Timing randomized clinical trial]**

**When to Take High Blood Pressure Medications: Morning or Night?**

Although exercise and other lifestyle changes can help lower blood pressure, many people require treatment with medicines. The most commonly recommended treatments are once-a-day blood pressure medications. These medicines work over the course of a day to help keep blood pressure under control and patients are told that they should take the medicine at the same time every day for best results.

However, it is not known whether taking antihypertensive medicines in the **morning** or at **night** is more effective. Because it is not known which is better, doctors usually do not give guidance on when it is best to take these medicines. Even if it turns out that one time is only slightly better than the other, because so many people have high blood pressure, this knowledge could benefit the long-term health of millions of people.

**Clinical trial proposal**

To show which of the two is better, a randomized clinical trial (this means that medicines will be randomized between participants) will be conducted.. This means that when each patient enters the study a computer program will randomly decide (like the flip of a coin) whether the patient should be told to take the medicine in the morning or at night. After one year, the patients who are told to take the medicine in the morning will be compared to the patients who are told to take the medicine at night to assess which treatment is better. The patient's medicine can be changed at any time if the patient or the patient’s doctor feels that a different medicine would be better.

**Recruiting Participants**

The research ethics committee is composed of physicians, nurses, individuals with no relation to health care and, at least, a lawyer and a patient representing the patients. Is tasked with overseeing all types of research conducted in the national health system. The research ethics committee s having a debate about the best way to conduct this trial.

Some members argue that patients should be given a **written consent** form about the study and asked to sign the form if they choose to participate. The consent form would **include information** about:

-the purpose of the research,

-risks and benefits,

-any alternative treatments,

-how patient privacy will be maintained,

-contact information for questions,

-a statement that participation is voluntary.

However, this written consent process will require extra time and effort from both the physician and the patient. As a result, requiring written consent would make it difficult to integrate research studies like this into routine health care practice. In some cases, if written consent is required, studies like these may not be done.

**[Option A; 2.1.General Information arm]**

Other members of the research ethics committee argue that written consent is unnecessarily burdensome in this case because the study has **very low risk**:

-doctors usually do not give guidance on what time of day it is best to take these medicines and

-they do not know when is better.

These members of the research ethics committee recommend that it is enough to provide **general notification** to all patients (through posters, brochures, and letters) that the hospital conducts research like this. Eligible patients for the trial who wish to begin treatment for their high blood pressure would automatically be enrolled in the trial without being informed or specifically asked if they would like to participate. From the patients’ perspective, all other aspects of care they receive would be no different than usual, except for the fact that a computer randomly chooses the treatment.

Q1c. If you were to give advice to the ethics review board, would you recommend Written Consent or General Notification?

| Written Consent | | General Notification | |
| --- | --- | --- | --- |
| Definitely | Probably | Probably | Definitely |
| 1 | 2 | 3 | 4 |

Q2c. If you were a patient in this hospital, which would you personally prefer, Written Consent or General Notification?

| Written Consent | | General Notification | |
| --- | --- | --- | --- |
| Definitely | Probably | Probably | Definitely |
| 1 | 2 | 3 | 4 |

**[Option B; 2.2. Verbal Consent arm]**

Other members of the research ethics committee argue that written consent is unnecessarily burdensome in this case because the study has **very low risk**:

-doctors usually do not give guidance on what time of day it is best to take these medicines and

-they do not know when is better.

These members of the research ethics committee recommend that it is enough to obtain **verbal consent** from the patient. The patient’s doctor would briefly explain the trial of taking antihypertensive medicines in the morning or at night:

-explain that it is not known which is better, and

-emphasize that the selection will be random.

The doctor would ask whether the patient would like to participate in the trial, and then record the patient’s decision in the medical record. For those that participate, all other aspects of care they receive would be no different than usual, except for the fact that a computer randomly chooses the time the treatment should be taken.

Q1d. If you were to give advice to the ethics review board, would you recommend Written Consent or Verbal Consent?

| Written Consent | | Verbal Consent | |
| --- | --- | --- | --- |
| Definitely | Probably | Probably | Definitely |
| 1 | 2 | 3 | 4 |

Q2d. If you were a patient in this health care system, which would you personally prefer, Written Consent or Verbal Consent?

| Written Consent | | Verbal Consent | |
| --- | --- | --- | --- |
| Definitely | Probably | Probably | Definitely |
| 1 | 2 | 3 | 4 |

**[To all respondents of Scenario 2; 2.3. To both scenarios 2.1.and 2.2]**

To finalize, we would also like to ask some questions about your opinions on how this proposed clinical trial compares to usual care. As a reminder:

A.In the randomized trial the patient’s doctor will use a computer to randomly choose when the treatment should be taken (Morning or Night) for the patient.

In usual care most doctors will not give directions on when to take the medication.  Some doctors may suggest taking the medication in the morning or at night.

In either case, the medication the patient is taking can be changed at any time if the patient or the patient’s doctor feels that a different medicine would be better.

Please rate the following statements on a scale from 1 (strongly disagree with the content of the sentence) to 7 (strongly agree).

| Strongly Disagree |  |  | I would not know to give an opinion |  |  | Strongly Agree |
| --- | --- | --- | --- | --- | --- | --- |
| 1 | 2 | 3 | 4 | 5 | 6 | 7 |

Q3a. It is valuable to study whether one treatment option is more effective than the other for treating high blood pressure.

Q4a. Patients who participate in the randomized trial face greater risks than patients who receive usual care.

Q5a. Patients who participate in the randomized trial are more likely to improve (lower) their high blood pressure than patients who receive usual care.

**We have concluded. Thanks for your collaboration**
