# Supplementary material for: Patients’ beliefs regarding informed consent for low-risk pragmatic trials
Source: BMC Med Res Methodol. 2017 Sep 18;17:145. doi: 10.1186/s12874-017-0424-3 (PMC5604493; doi:10.1186/s12874-017-0424-3)
Supplement: Supplementary file 2 — Recommendation to research ethics committee and personal preference: written consent or alternative option. Logistic regression (DOCX 14 kb) [file 12874_2017_424_MOESM2_ESM.docx]

**Dal-Ré, Carcas, Carné & Wendler**

**Additional file-2.**

Recommendation to research ethics committee and personal preference: written consent or alternative option. Logistic regression

| ***Recommendation to research ethics committee*** | ***% **** | ***Odds Ratio*** | ***P value*** |
| --- | --- | --- | --- |
| **Scenario / option** |  |  |  |
| *Drug pRCT*  Written consent vs General Notification. | 31.8 | 2.910 | **<0.001** |
| Written consent vs Verbal Consent.  *Dose timing pRCT* | 17.6 | 1.117 | 0.553 |
| Written consent vs General Notification. | 40.0 | 3.712 | **<0.001** |
| Written consent vs Verbal Consent. | 13.3 | 1 |  |
| * Percentage of respondents recommending the alternative option | | | |

| ***Personal preference*** | ***% **** | ***Odds Ratio*** | ***P value*** |
| --- | --- | --- | --- |
| **Scenario / option** |  |  |  |
| *Drug pRCT*  Written consent vs General Notification. | 30.6 | 2.724 | **<0.001** |
| Written consent vs Verbal Consent.  *Dose timing pRCT* | 15.3 | 1.061 | 0.752 |
| Written consent vs General Notification. | 44.7 | 3.974 | **<0.001** |
| Written consent vs Verbal Consent. | 13.3 | 1 |  |
| * Percentage of respondents preferring the alternative option | | | |

pRCT: Pragmatic randomized controlled trial
